# Neuroleptic malignant syndrome and serotonin syndrome: a comparative bibliometric analysis

**DOI:** 10.1186/s13023-024-03227-5

**Published:** 2024-06-02

**Authors:** Waleed M. Sweileh

**Affiliations:** Department of Biomedical Sciences, Faculty of Medicine and Health Sciences, An-Najah National University, Nablus, Palestine

**Keywords:** Neuroleptic malignant syndrome, Serotonin syndrome, Research publications, comparative analysis

## Abstract

**Objective:**

This study aimed to analyze and map scientific literature on Neuroleptic Malignant Syndrome (NMS) and Serotonin Syndrome (SS) from prestigious, internationally indexed journals. The objective was to identify key topics, impactful articles, prominent journals, research output, growth patterns, hotspots, and leading countries in the field, providing valuable insights for scholars, medical students, and international funding agencies.

**Methods:**

A systematic search strategy was implemented in the PubMed MeSH database using specific keywords for NMS and SS. The search was conducted in the Scopus database, renowned for its extensive coverage of scholarly publications. Inclusion criteria comprised articles published from 1950 to December 31st, 2022, restricted to journal research and review articles written in English. Data were analyzed using Microsoft Excel for descriptive analysis, and VOSviewer was employed for bibliometric mapping.

**Results:**

The search yielded 1150 articles on NMS and 587 on SS, with the majority being case reports. Growth patterns revealed a surge in NMS research between 1981 and 1991, while SS research increased notably between 1993 and 1997. Active countries and journals differed between NMS and SS, with psychiatry journals predominating for NMS and pharmacology/toxicology journals for SS. Authorship analysis indicated higher multi-authored articles for NMS. Top impactful articles focused on review articles and pathogenic mechanisms. Research hotspots included antipsychotics and catatonia for NMS, while SS highlighted drug interactions and specific medications like linezolid and tramadol.

**Conclusions:**

NMS and SS represent rare but life-threatening conditions, requiring detailed clinical and scientific understanding. Differential diagnosis and management necessitate caution in prescribing medications affecting central serotonin or dopamine systems, with awareness of potential drug interactions. International diagnostic tools and genetic screening tests may aid in safe diagnosis and prevention. Reporting rare cases and utilizing bibliometric analysis enhance knowledge dissemination and research exploration in the field of rare drug-induced medical conditions.

## Introduction

Neuroleptic malignant syndrome (NMS) and serotonin syndrome (SS) are drug-induced, potentially life-threatening conditions that are infrequently encountered in medical practice, necessitating prompt intervention [1–4]. Neuroleptic Malignant Syndrome is characterized by a decrease in dopamine activity in the brain, often associated with the use of dopamine antagonists, primarily neuroleptic or antipsychotic medications [5, 6]. While the exact pathophysiology of NMS remains incompletely understood, it is believed to involve dopamine dysregulation in the basal ganglia and hypothalamus. This dysregulation, particularly the blockade of dopamine receptors, especially D2 receptors, leads to a state of dopamine deficiency, manifesting in symptoms such as muscle rigidity, hyperthermia, and autonomic instability. Furthermore, withdrawal from dopamine agonists, such as L-Dopa, can also precipitate NMS in susceptible individuals. Serotonin Syndrome is characterized by an excess of serotonin (5-HT) in the central nervous system, typically stemming from the use of serotonergic medications or substances that elevate serotonin levels [7, 8]. These drugs encompass antidepressants, notably selective serotonin reuptake inhibitors (SSRIs), opioids, specific psychedelics, serotonin agonists, and herbal supplements. The pathophysiology of SS revolves around the excessive stimulation of serotonin receptors, particularly the 5-HT2A receptors. This heightened stimulation precipitates a spectrum of symptoms, ranging from agitation, confusion, hyperthermia, muscle rigidity, to autonomic dysfunction. The severity of SS can vary widely, from mild manifestations to life-threatening conditions, contingent upon the extent of serotonin excess and individual susceptibility factors.

Both NMS and SS exhibit shared clinical manifestations, including hyperthermia, hypertension, hypersalivation, diaphoresis, and altered mental status [4], with instances of coexistence reported in some patients [9]. However, they diverge in their etiologies and clinical presentations. For instance, individuals with NMS typically display hyporeflexia, normal pupil size, and normal bowel sounds, contrasting with SS patients who often present with hyperreflexia, dilated pupils, and hyperactive bowel activity [10]. NMS is typified by lead-pipe muscle rigidity, whereas SS manifests with increased muscle tone, particularly in the lower extremities [11, 12]. Given these distinctions, treatment strategies for NMS and SS diverge based on their underlying causes [2]. The mechanisms driving these syndromes differ significantly; while NMS involves diminished dopamine activity in the brain, SS is characterized by elevated serotonin levels [13]. Dopamine antagonists, such as neuroleptics or antipsychotics, are commonly implicated in NMS [14–16], although other triggers like withdrawal from dopamine agonists, like L-Dopa, can also induce NMS [17, 18]. Conversely, SS can result from various drug classes, including antidepressants, opioids, psychedelics, serotonin agonists, and certain herbs [7, 19–23]. Consequently, distinct medications are employed for their management; benzodiazepines and serotonin antagonists are standard therapy for SS, whereas dopaminergic agents and dantrolene are preferred for NMS [10]. While the incidence of NMS remains low, particularly among patients receiving newer generation antipsychotics [24, 25], recent studies on SS incidence are lacking. However, a 1999 study reported an incidence of 0.4 cases per 1000 patient-months with nefazodone [26], while SS incidence reaches 14–16% in cases of selective serotonin reuptake inhibitor (SSRI) overdose [27].

### Research context and objectives

The landscape of psychiatric pharmacotherapy has evolved over time, witnessing a surge in the number of approved drugs and the introduction of novel classes into clinical practice [28–31]. This trend is particularly notable in the treatment of depression and schizophrenia, where the absence of universally safe and effective drugs persists [32–36]. Additionally, off-label utilization of antidepressants and antipsychotics has been observed among patients with dementia and other neuro-cognitive disorders [37–41], contributing to an upward trajectory in psychiatric drug consumption [42, 43]. The risk of SS is linked to any medication or herb augmenting the central serotonergic pathway, necessitating vigilant monitoring by healthcare professionals due to the potential for adverse effects, whether as a primary mechanism or side effect [20]. A concerning trend of unsupported polypharmacy in psychiatric medications has also emerged [44], along with significant prescribing of antidepressants and antipsychotics to dementia patients without documented indications of depression or psychosis [45, 46], mirroring similar trends among individuals with intellectual disabilities [47]. The escalating demand for psychiatric therapy raises apprehensions regarding the likelihood of adverse medication effects [48], exacerbated by increased prescribing rates, polypharmacy, and off-label usage, which heighten the incidence of drug-induced toxicities, including NMS and SS. Analyzing published literature on drug-induced NMS and SS provides valuable insights into these rare yet severe toxicities, aligning with the pressing global public health burden of depression, schizophrenia, and related conditions, accentuated by the fatal toxicities associated with specific psychiatric medications. This scientific literature on NMS and SS is ripe for analysis and mapping to delineate current research hotspots [49–55], addressing the gap in the literature. Accordingly, the present study aims to analyze and map scientific research on NMS and SS published in prestigious, internationally indexed journals. Through this analysis, the study seeks to identify key topics, impactful articles, prominent journals, research output, growth patterns, hotspots, and leading countries in the field, providing valuable insights for scholars, medical students, and international funding agencies to discern research trajectories, bibliographic trends, and knowledge structures pertaining to NMS and SS. Ultimately, this endeavor aims to invigorate scholarly discourse and inform clinical practice in the field.

## Methods

### Database and keywords

In this study, we employed a systematic search strategy to extract relevant scientific literature on NMS and SS from the PubMed MeSH database. Specifically, we utilized the following keywords:

Malignant neuroleptic syndrome: “malignant neuroleptic syndrome”.

Serotonin syndrome: “serotonin syndrome” or “serotonin toxicity”.

To ensure comprehensive coverage, we conducted our search in Scopus, a prestigious scientific database owned by Elsevier, which has previously been utilized for analyzing research in psychiatry [56, 57]. Scopus is renowned for its extensive coverage, encompassing a vast array of scholarly publications in the field. Notably, Scopus encompasses over 95% of the content included in other databases such as PubMed and Web of Science, rendering it an ideal platform for our study [58].

### Inclusion and exclusion criteria

We restricted our search to articles published from 1950 to December 31st, 2022, and focused exclusively on journal research and review articles written in English. Excluded from our analysis were editorials, notes, letters, and conference abstracts. Additionally, articles pertaining to non-human subjects were excluded, ensuring the relevance of our findings. We meticulously reviewed the titles and abstracts of over 100 articles to eliminate irrelevant publications, such as those mentioning NMS or SS only marginally, thereby refining the scope of our analysis.

### Validation

Our search strategy yielded results indicative of its validity, as evidenced by the prominent presence of leading scientists and journals in the fields of psychiatry and pharmacology. This reaffirmed the robustness of our search criteria and the relevance of the retrieved literature to our study objectives.

### Data management, analysis, and mapping

The dataset comprising the retrieved articles was subjected to descriptive analysis using Microsoft Excel. Subsequently, we employed VOSviewer, a freely available online tool, for bibliometric mapping purposes [59]. VOSviewer maps offer researchers a visual tool for exploring bibliometric data, revealing patterns, relationships, and trends within a dataset. Interpretation of these maps involves understanding several key elements. Firstly, node size indicates the prominence or frequency of an item, with larger nodes representing more significant themes or influential publications. Secondly, node color categorizes items into clusters, with similar colors indicating thematic groupings. Thirdly, the thickness of connecting lines between nodes signifies the strength of associations, with thicker lines indicating stronger connections. Lastly, the distance between nodes reflects the similarity or dissimilarity between items, with closer nodes indicating stronger relationships. Overall, VOSviewer maps provide a comprehensive visual overview of bibliometric data, enabling researchers to identify clusters, influential publications, and emerging trends within their field of study by considering the interplay between node size, color, line thickness, and spatial relationships. Within the descriptive analysis, we presented lists of active countries and journals, alongside a linear graph illustrating the growth of publications over time. In the keyword visualization map generated using VOSviewer, node size corresponded to the frequency of occurrence of each keyword, enabling visual identification of prominent themes. Similarly, in the journal visualization map, node size reflected the normalized citation count received by each journal, providing insights into publication impact within the field.

## Results

### Number of publications

The search strategy yielded a total of 1150 articles on NMS and 587 on SS. Among the articles on NMS, 791 (68.8%) were case reports, while 384 (65.4%) of the articles on SS took the form of case reports.

### Growth of publications

**Fig. 1:**
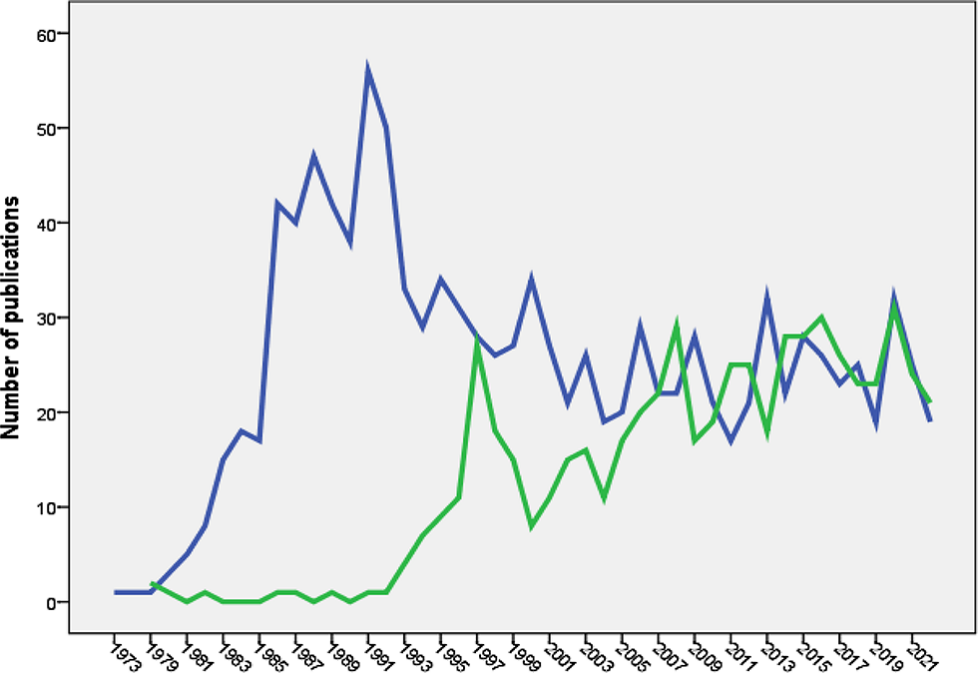
Annual growth of publications of NMS (blue line) and SS (green line). The Figure was created by SPSS program

The earliest scientific publication on NMS dates back to 1973 [60], while publications on SS emerged in 1979 [61]. Research on NMS experienced a notable surge between 1981 and 1991, followed by a fluctuating decline. Conversely, research on SS saw a steep increase between 1993 and 1997, followed by a fluctuating rise. Figure 1 illustrates the growth trends of research on NMS and SS.

### Active countries and journals

Table 1 outlines the top five countries contributing articles on NMS and SS. Japan ranked second in NMS publications but fifth in SS publications. Table 2 presents the top five active journals for both NMS and SS, with NMS publications primarily within psychiatry journals and SS publications within pharmacology/toxicology journals.

**Table 1:** Top five countries publishing articles on NMS or SS

Active countries in publishing articles on NMS	Number (%) *N* = 1150	Active countries in publishing articles on SS	Number (%) *N* = 587
United States	430 (37.4)	United States	291 (49.6)
Japan	118 (10.3)	United Kingdom	45 (7.7)
United Kingdom	89 (7.7)	Australia	40 (6.8)
India	60 (5.2)	India	30 (5.1)
Canada	49 (3.2)	Canada	21 (3.6)
-	-	Japan	21 (3.6)

NMS = Neuroleptic malignant syndrome

SS = Serotonin syndrome/ Serotonin toxicity

### Authorship analysis

Articles on NMS involved 3820 authors (mean = 3.1 authors per article), with 89 (7.3%) single-authored and 171 (14.1%) multi-authored articles. Similarly, articles on SS included 2105 authors (mean = 3.0 authors per article), with 102 (16.0%) single-authored and 41 (7.1%) multi-authored articles.

### Most impactful articles

The top five impactful articles on NMS comprised mainly review articles and a research article focusing on the pathogenic role of dopamine antagonists [62]. For SS, the top five impactful articles included review articles and research articles discussing the Hunter diagnostic criteria [63] and the role of monoamine oxidase inhibitors (MAO-I) and opioid analgesics in serotonin toxicity [64].

### Research hotspots

**Fig. 2:**
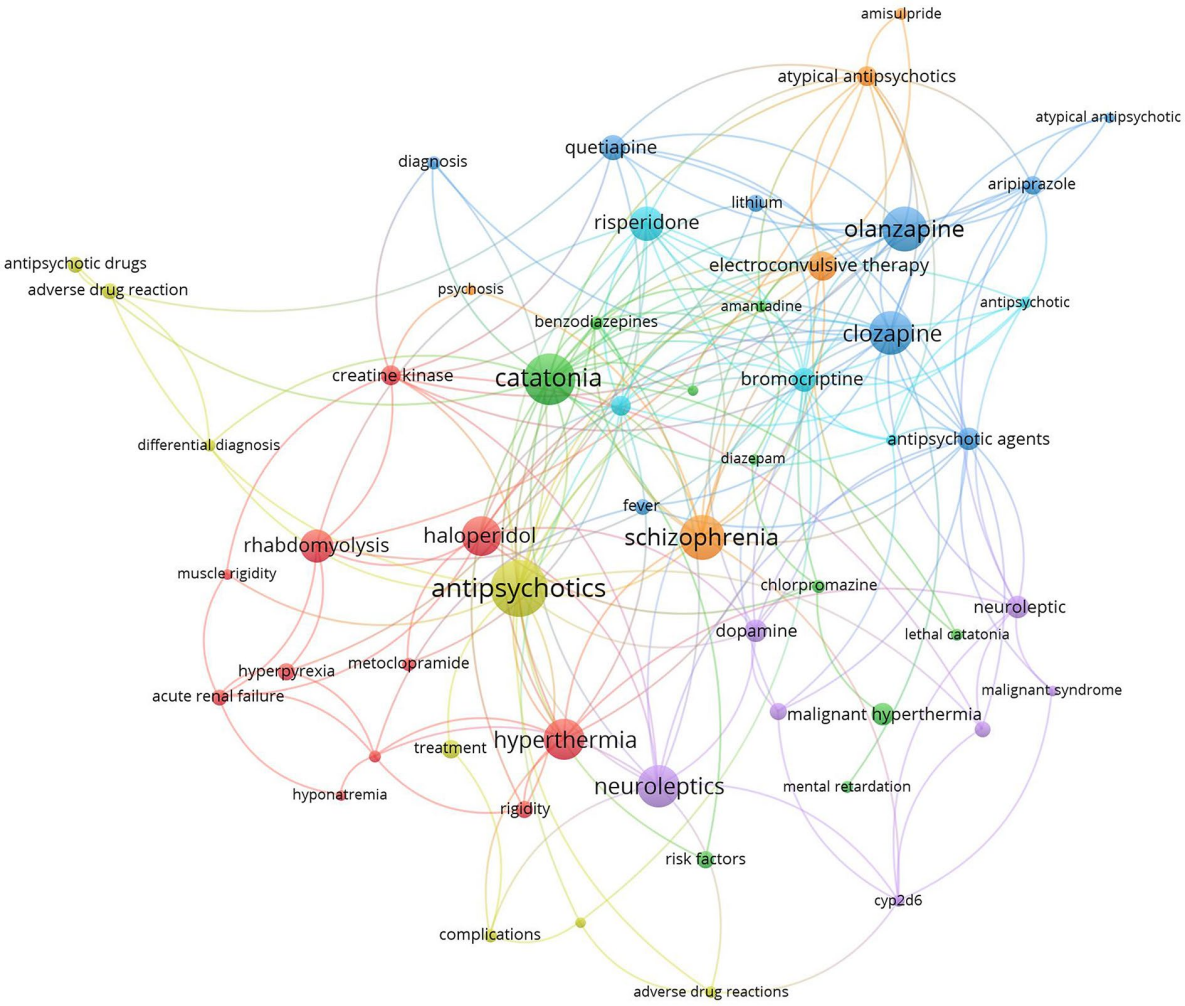
Network visualization map of author keywords with minimum occurrences of five times. Large nodes represent research hotspots on NMS. The term NMS was not shown to make other keywords more visible

**Fig. 3:**
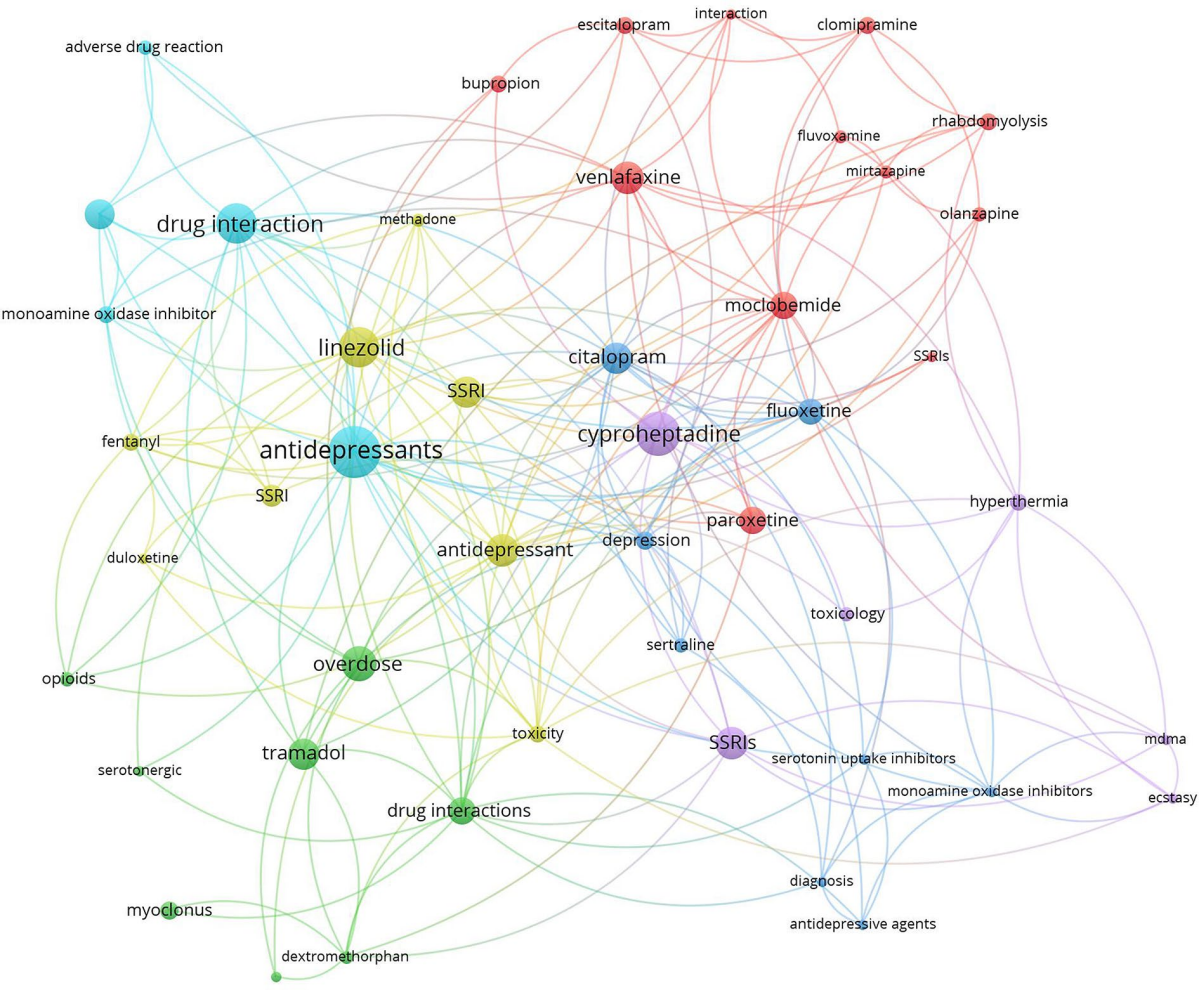
Network visualization map of author keywords with minimum occurrences of five times. Large nodes represent research hotspots on SS. The term SS was not shown to make other keywords more visible

Research hotspots were identified by mapping author keywords with a minimum occurrence of five times (Figs. 2 and 3). Notable hotspots for SS included antidepressants, SSRIs, tramadol, linezolid, cyproheptadine, and drug interactions. For NMS, hotspots included antipsychotics (various drug names), catatonia, and rhabdomyolysis.

### Journal citation analysis

**Fig. 4:**
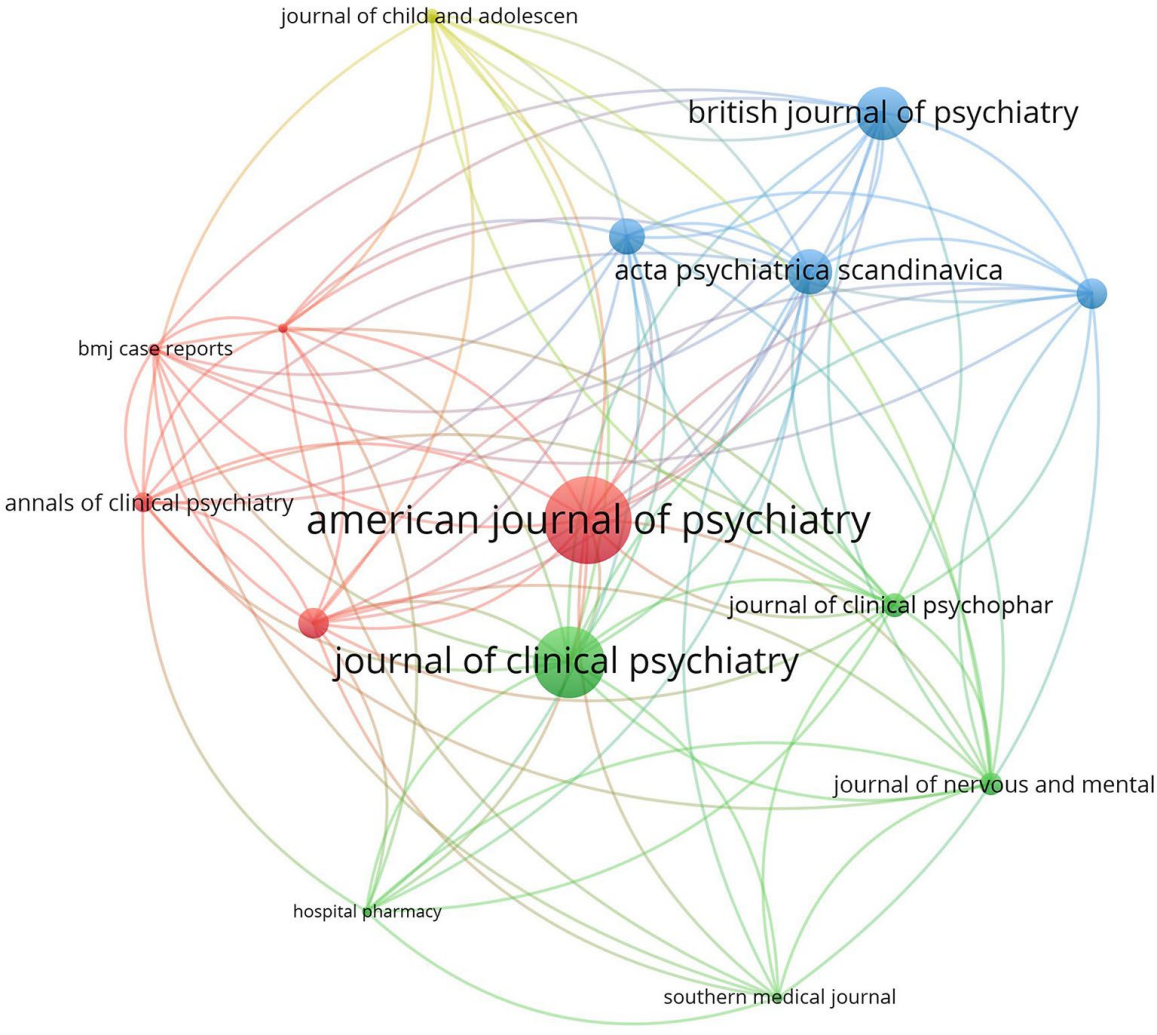
Network visualization map of the top 15 journals in the field of NMS. Large node sized indicates higher normalized citation count

**Fig. 5:**
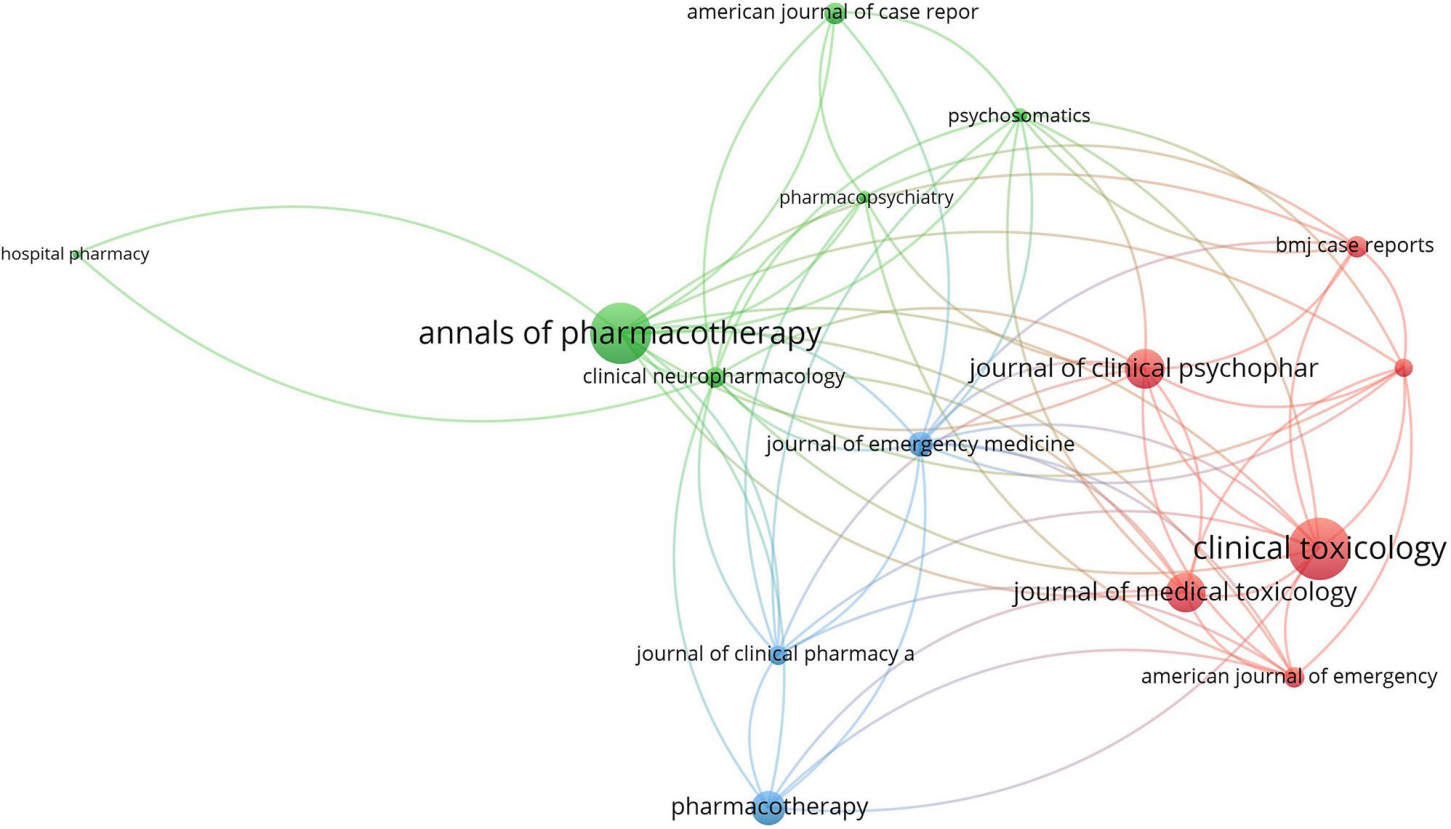
Network visualization map of the top 15 journals in the field of SS. Large node sized indicates higher normalized citation count

The top 15 active journals in publishing articles on NMS and SS were mapped (Figs. 4 and 5). Notably, articles on NMS published in the *American Journal of Psychiatry* and the *Journal of Clinical Psychiatry* received the highest number of citations per article. Similarly, articles on SS published in *Clinical Toxicology* and *the Annals of Pharmacotherapy* garnered the most citations per article.

### Geographic mapping

**Fig. 6:**
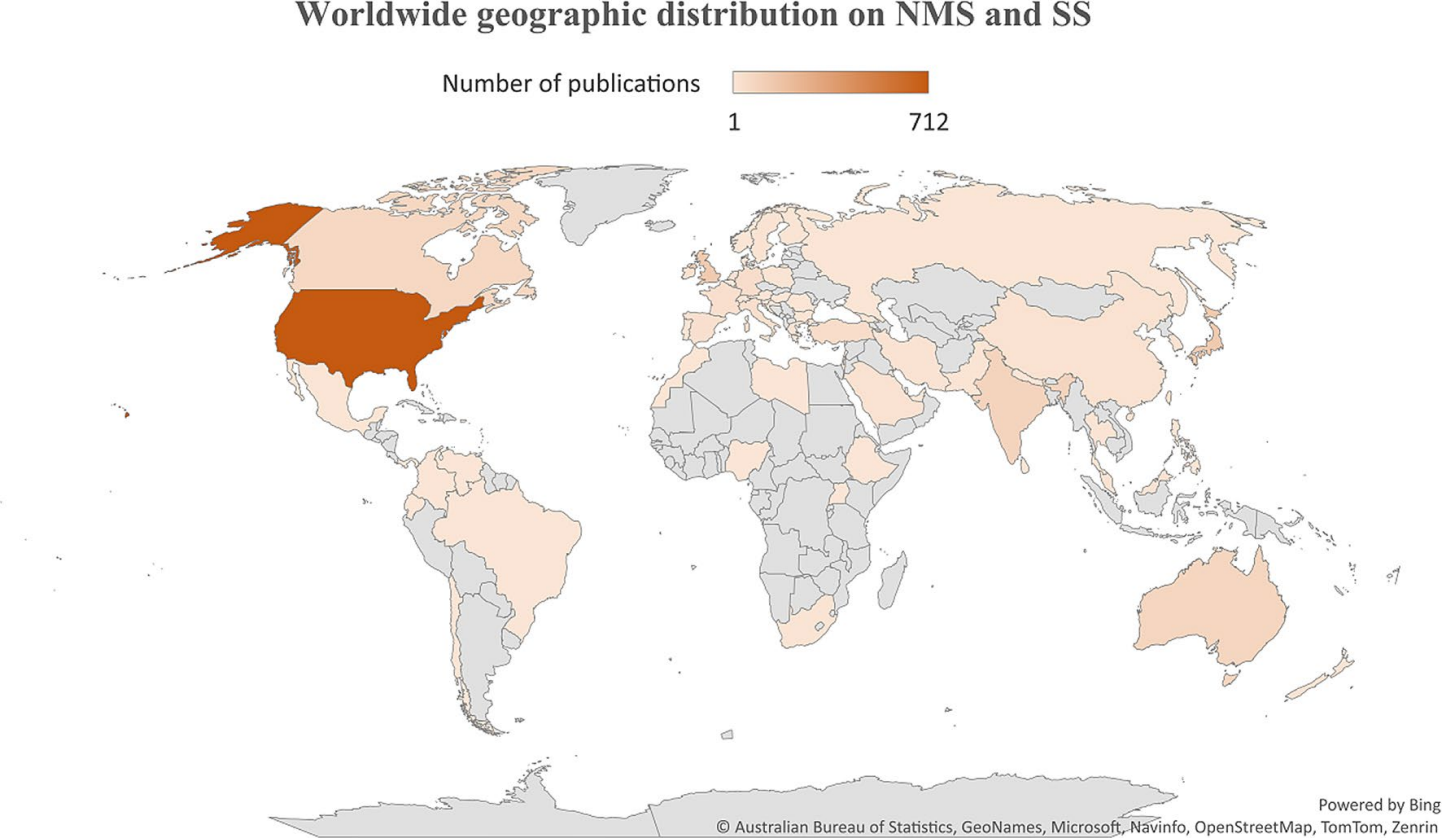
Worldwide distribution of research publications on NMS and SS. Figure was created by Microsoft Excel

The geographic distribution of research publications on NMS and SS was illustrated on a worldwide map (Fig. 6), with the majority of contributions originating from the US. Several countries in specific regions showed minimal to no research output on either NMS or SS.

### Molecular genetics

The retrieved literature on NMS has 20 articles that discussed the potential link between NMS and certain genetics. Ten articles discussed the potential linkage between Cytochrome 2D6 and potential risk for NMS [65–74]. Five articles discussed the potential linkage between dopamine receptor 2 gene polymorphism and NMS [75–79]. Four articles discussed the linkage between ryanodine receptor gene mutations and susceptibility to NMS [80–83]. No association was found between NMS and serotonin receptor gene variation [84]. The literature on SS has few articles that discussed the genetic predisposition of patients to SS such as the 5-HT receptor gene or the CYP 2D6 gene polymorphism [85, 86].

### Drug interactions

Serious drug-drug interactions leading to NMS were mentioned in a limited number of articles and involved the administration of two dopamine antagonists [87] or two atypical antipsychotic drugs [88]. However, there were many articles discussing potential SS caused by drug-drug interactions, which included SSRI–methylene blue [89], SSRI–metoclopramide [89], sertraline–phenelzine [90], anti-depressants–opioids [91], citalopram-fentanyl [92], a combination of two anti-depressants [93], SSRI-linezolid [94–102], sertraline–phenelzine [90], citalopram-buspirone [103], venlafaxine-tranylcypromine [104], and many others [92, 105–109].

### Non-psychiatric causative agents

The retrieved literature on SS, showed that several drugs and drug classes not related to antidepressants can induce SS. Examples of such drugs included Linezolid, CNS stimulants (amphetamine), hallucinogens (LSD), opioids (fentanyl), ondansetron, sumatriptan, and certain herbs (St. John’s wort), metoclopramide, ritonavir, and others [5, 20, 110, 111]. The retrieved literature on NMS showed that drug-induced NMS is limited to antipsychotics and withdrawal of dopamine agonists [112–114].

### Diagnostic criteria

For NMS, there were 30 articles that discussed issues related to diagnosis. In 2011, an international panel tried to develop NMS diagnostic criteria [115, 116]. The neutrophil-lymphocyte ratio was suggested by certain researchers as a diagnostic test for NMS [117, 118]. The differential diagnosis for NMS compared to SS and catatonia was also published [13, 118, 119]. For SS, there were 17 articles that discussed issues related to diagnosis of SS. The Hunter diagnostic criteria was one of these articles [63]. Other articles discussed controversies and the importance of differential diagnosis in SS [120].

## Discussion

The current study analyzed and compared the scientific literature on two rare drug-induced conditions with certain overlapping clinical features. Both syndromes are mainly caused by medications used in psychiatry, such as those for schizophrenia and depression. The name “NMS” implies that the syndrome is correlated with the use of neuroleptic medications, while the name “SS” implies that it is correlated with any medication or herb that raises serotonin centrally.

The analysis showed that the volume of research publications on NMS was larger and started earlier than research publications on SS. The NMS is associated with the use of dopamine antagonists (neuroleptics). The history of using old-generation antipsychotics for the treatment of schizophrenia dates back to the 1950s [121–125]. On the other hand, the introduction of the SSRI drug class, the main causative agent of SS, dates back to the late 1980s [126]. The difference in the history of introduction into clinical practice explains the differences between SS and NMS in growth patterns. The difference in the volume of literature between the two syndromes could be due to diagnostic uncertainty [127] for NMS versus SS, the seriousness of medical complications, or debate regarding whether an atypical antipsychotic drug class causes NMS in a similar way to conventional antipsychotics [13, 30, 128–130]. The current study showed that the number of research publications on NMS started to decline after 1991 but the number of publications on SS started to increase after 1997. The introduction of atypical antipsychotics with lesser dopaminergic side effects than conventional antipsychotics decreased the incidence of NMS and therefore decreased the number of publications with time. On the other hand, the increased number of SS publications after 1997 could be explained by the many reported drug interactions at serotonin level leading to more cases of SS with time.

The current study showed that journals in the field of psychiatry ranked highest in publishing articles on NMS, while those in the field of pharmacology/toxicology ranked highest in publishing articles on SS. The reason for this difference is difficult to explain. However, NMS is primarily limited to schizophrenia patients taking antipsychotic drugs, while SS might occur in normal people taking SSRIs for depression or any other condition. Furthermore, the potentially large numbers of drug- or drug-herb interactions make the SS interesting to pharmacology/toxicology journals [22]. Actually, SS has been termed “serotonin toxicity” implying relatedness to toxicology [131].

The findings of the current study regarding active countries were not surprising. The English-speaking countries, the US, the UK, Australia, and Canada showed leading roles in many scientific disciplines and ranked first in several studies that analyzed research activity [132–135]. This is due to advancements in technology, medicine, clinical practice, and research funding in high-income countries relative to other countries. However, there are also reasons related to the nature of journals indexed in Scopus. The vast majority of Scopus-indexed journals publish articles in English, and the vast majority of the journals are issued by publishers and institutions based in the US, Europe, or Australia. Therefore, Scopus might be biased toward scholars in English-speaking countries. The finding that research articles on NMS tend to be multi-authored while those on SS are not is not easy to explain. However, it is possible that cases of NMS tend to involve a larger medical team due to the nature of complications that might involve renal and blood complications. Furthermore, the treatment of NMS requires medications and follow-up. All this makes the number of authors in a case study of NMS higher than those involved in SS cases [13, 136, 137].

Of the retrieved articles on SS and NMS, the research *article “The hunter serotonin toxicity criteria: Simple and accurate diagnostic decision rules for serotonin toxicity”* [63] received the highest number of citations excluding the review articles. The diagnosis of SS is based on the clinical symptoms and the medical history of the patient. Harvey Sternbach introduced the first diagnostic criteria for SS in 1991 and the *Hunter Diagnostic Criteria* tool was introduced in 2003 [63, 138].

Mapping the retrieved literature on NMS showed that rhabdomyolysis and catatonia constituted distinct research hotspots in addition to those related to antipsychotic medications and schizophrenia. However, mapping the author keywords of SS research publications showed that linezolid, drug interactions, and tramadol constituted research hotspots in addition to antidepressants and SSRIs. Rhabdomyolysis has been reported as a consequence of NMS even among children and adolescents [139, 140]. However, reports of rhabdomyolysis among patients taking antipsychotics were published, suggesting that rhabdomyolysis could be a side effect of antipsychotics even in the absence of NMS [139, 140]. Catatonia is, as NMS, a consequence of neuroleptic drugs, and there is an overlap in clinical features between the symptoms of catatonia and those of NMS, which makes the distinction between them difficult [141]. Linezolid is an antibiotic that was originally designed to be used as an anti-depressant by virtue of its MAO enzyme inhibition property [142]. This explains the many cases of SS induced by drug interactions with Linezolid [141]. The relatively higher number of research articles on drug/herb interactions leading to SS is attributed to the presence of many and different drug classes that affect and increase serotonergic pathways in the brain [90, 105, 109, 143]. The scientific controversy about the potential ability of tramadol to cause SS received a high number of citations. Current scientific evidence supports the ability of tramadol to cause SS due to its molecular pharmacological effects on both the opioid and serotonergic systems [105, 107, 144–147]. Cyproheptadine was also a research hotspot in the field of SS. Cyproheptadine has anti-histaminic, anticholinergic, and anti-serotonergic properties and that is why it has been used to counter the symptoms of SS [148–150].

The current study showed that SS has a wide range of possible drug/herb interactions due to the many drugs that affect the serotonin system. Of particular interest is the one with opioid analgesics, since they are commonly used in hospital settings. Opioids, including fentanyl and even dextromethorphan in cough syrups, were reported to increase serotonin levels, and therefore caution should be practiced when given to patients with SSRIs in their medical records [19, 22, 109, 151].

**Table 2:** Top five journals publishing articles on NMS or SS

Active journals in publishing articles on NMS	Number (%) *N* = 1150	Active journals in publishing articles on SS	Number (%) *N* = 587
*British Journal of Psychiatry*	29 (2.5)	*Annals of Pharmacotherapy*	19 (3.2)
*Journal of Clinical Psychiatry*	27 (2.3)	*BMJ Case Reports*	15 (2.6)
*American Journal of Psychiatry*	21 (1.8)	*Clinical Toxicology*	13 (2.2)
*Biological Psychiatry*	21 (1.8)	*Hospital Pharmacy*	13 (2.2)
*Acta Psychiatrica Scandinavica*	18 (1.6)	*Clinical Neuropharmacology*	12 (2.0)

### Limitations

Limitations arise in this study from various factors. Firstly, the reliance on the Scopus database for literature retrieval could potentially limit the inclusivity of articles from low- and middle-income countries. Although Scopus offers extensive coverage, the possibility exists that some relevant journals from these regions may not be indexed, thereby leading to a potential underestimation of publications from certain geographic areas. Secondly, despite efforts to employ a comprehensive search strategy, the use of a title-abstract search method might have resulted in the retrieval of some false-positive results. While validation tests were conducted to mitigate this issue, the possibility of false positives cannot be entirely ruled out. Thirdly, the analysis focused solely on articles published in English-language journals, which could introduce a language bias and limit the generalizability of findings. This exclusion of literature published in other languages may have led to the omission of relevant data from non-English sources. Lastly, diagnostic uncertainty poses a challenge in distinguishing between neuroleptic malignant syndrome (NMS) and serotonin syndrome (SS) due to overlapping clinical features and the absence of definitive diagnostic tests. Misdiagnosis or underreporting of cases may have occurred, potentially impacting the accuracy of the literature analysis and conclusions drawn from it.

## Conclusions

In conclusion, NMS and SS represent rare but potentially life-threatening conditions associated with drug-induced dysregulation of dopamine and serotonin systems, respectively. The study analyzed and compared the scientific literature on these syndromes, revealing distinct growth patterns, research hotspots, and publication trends. The findings underscored the evolving landscape of psychiatric pharmacotherapy and the complexities involved in diagnosing and managing NMS and SS. While NMS research exhibited a longer history and a decline in publications over time, SS research witnessed a notable increase in publications, reflecting advancements in pharmacological understanding and the recognition of SS as a significant clinical entity. Identified research hotspots provided valuable insights into emerging areas of interest, including drug interactions, molecular genetics, and diagnostic criteria. Understanding these trends is essential for informing clinical practice, guiding future research endeavors, and promoting collaboration among scholars and healthcare professionals. Despite the study’s contributions, several limitations warrant consideration, including database restrictions, potential publication bias, and diagnostic uncertainties. Addressing these limitations through expanded literature search strategies, international collaboration, and improved diagnostic tools is crucial for advancing knowledge and enhancing patient care in the field of rare drug-induced syndromes. Moving forward, efforts to develop standardized diagnostic criteria, genetic screening tools, and international reporting mechanisms for NMS and SS are warranted. Additionally, continued bibliometric analysis and mapping of literature on rare medical conditions can facilitate ongoing research and contribute to the dissemination of knowledge across global healthcare communities.

## Data Availability

All data present in this article can be retrieved from Scopus using keywords listed in the methodology.

## Abbreviations

NMS: Neuroleptic malignant syndrome
SS: Serotonin Syndrome/ Serotonin toxicity
